# Autofluorescence of Model Polyethylene Terephthalate Nanoplastics for Cell Interaction Studies

**DOI:** 10.3390/nano12091560

**Published:** 2022-05-04

**Authors:** Francesca Lionetto, Maria Giulia Lionetto, Claudio Mele, Carola Esposito Corcione, Sonia Bagheri, Gayatri Udayan, Alfonso Maffezzoli

**Affiliations:** 1 Department of Engineering for Innovation, University of Salento, Via per Monteroni, 73100 Lecce, Italy; claudio.mele@unisalento.it (C.M.); carola.corcione@unisalento.it (C.E.C.); sonia.bagheri@unisalento.it (S.B.); alfonso.maffezzoli@unisalento.it (A.M.); 2 Department of Biological and Environmental Sciences and Technologies (DISTEBA), University of Salento, Via per Monteroni, 73100 Lecce, Italy; giulia.lionetto@unisalento.it (M.G.L.); gayatri.udayan@unisalento.it (G.U.)

**Keywords:** label-free nanoplastics, model nanoplastics, nanoplastic internalization, polyethylene terephthalate, autofluorescence, ocean pollution, atomic force microscopy

## Abstract

This work contributes to fill one of the gaps regarding nanoplastic interactions with biological systems by producing polyethylene terephthalate (PET) model nanoplastics, similar to those found in the marine environment, by means of a fast top-down approach based on mechanical fragmentation. Their size distribution and morphology were characterized by laser diffraction and atomic force microscopy (AFM). Their autofluorescence was studied by spectrofluorimetry and fluorescence imaging, being a key property for the evaluation of their interaction with biota. The emission spectra of label-free nanoplastics were comparable with those of PET nanoplastics labeled with Nile red. Finally, the suitability of label-free nanoplastics for biological studies was assessed by in vitro exposure with Mytilus galloprovincialis hemolymphatic cells in a time interval up to 6 h. The nanoplastic internalization into these cells, known to be provided with phagocytic activity, was assessed by fluorescence microscopy. The obtained results underlined that the autofluorescence of the model PET nanoplastics produced in the laboratory was adequate for biological studies having the potential to overcome the disadvantages commonly associated with several fluorescent dyes, such as the tendency to also stain other organic materials different from plastics, to form aggregates due to intermolecular interactions at high concentrations with a consequent decrease in fluorescence intensity, and to dye desorption from nanoparticles. The results of the autofluorescence study provide an innovative approach for plastic risk assessment.

## 1. Introduction

Ocean pollution by anthropogenic waste has been recognized as a serious global environmental problem worsened by the presence of microplastics (MPs) and nanoplastics (NPs), generally classified as polymer particles with size lower than 1 μm and 5 mm, respectively [1,2]. NPs are less understood than MPs due to the lack of suitable analytical methods, but, at the same time, NPs are suspected of having the greatest impact on the environment and living organisms [3]. Increasing concerns about NPs arise from their very small size, their easy transport in the environment, their sorption and release of pollutants, and their biomagnification effect through the food chain up to humans [4,5]. Several in vitro and in vivo studies have shown that MPs and NPs may cause impacts on the human body, including physical stress and damage, apoptosis, necrosis, inflammation, oxidative stress, and immune responses, as recently reviewed [6,7]. Bridging the knowledge gap about NPs can be considered fully part of the Goal 14 “Life below water” target of the 2030 Agenda for Sustainable Development, promoted by United Nations [8] relative to the increase of the scientific knowledge, research, and technology for ocean health.

NPs have been recently detected in aquatic environments, but not yet quantified, since their sampling, extraction from environmental matrices, and characterization is technically very difficult due to the limited signals resulting from low volumes and masses [3,9,10]. Therefore, the evaluation of environmental fate and impact of nanoplastics on living organisms and human health is very challenging and is actually carried out using simplified model nanoplastics. These latter are mainly monodisperse spherical polystyrene (PS) nanoparticles, often obtained via water-dispersed processes with surfactants and preservatives, as reported by El Hadri et al. [11]. They have limited environmental relevance, being characterized by physicochemical properties and shapes different from those related to MPs/NPs found in environmental samples [12,13,14]. This raises several questions about the realistic environmental impact of PS NPs. As reported by Rowenczyk et al. [15], the composition of model NPs and their stability and concentration are the main factors that could increase the environmental relevance of many studies.

Due to their extensive production, use, and mismanagement, different micro- and nanosized thermoplastic polymers can be found in the environment, such as polyethylene (PE), polyethylene terephthalate (PET), and polypropylene (PP) [6]. In particular, PET MPs/NPs have been sampled and reported [16,17,18,19], but their hazardous effect to environment and human health remain largely unknown. Very recently, Aguilar-Guzman et al. [20] demonstrated that PET NPs can be easily internalized by mouse macrophages cells. More recently, Leslie et al. [21] discovered and quantified PET NPs in human blood, demonstrating their bioavailability for uptake into the human bloodstream.

The laboratory-made or commercially available NPs are then labeled with fluorescent dyes in order to be clearly detectable when they are used in biological studies. A great number of studies are reported in the literature on the use of fluorescent-dye-labeled micro/nanoplastics in toxicological and eco-toxicological studies addressed to investigate micro/nanoplastics interactions with biological systems. However, the possibility to eliminate the fluorescent-dye-labeling step on MPs/NPs could be very useful since, besides the expected time and cost saving, the drawbacks associated mostly with non-specific fluorescence background, false positives, and to the instability of the photophysical properties of fluorophores, i.e., photobleaching at prolonged exposures, could be avoided [22,23]. Moreover, the chemical nature of the dye could affect the micro/nanoplastic behavior in eco-toxicological studies, or the dye can be leached and absorbed by studied organisms, leading to misleading results.

An alternative approach to fluorescence labeling of MPs and NPs could be the exploitation of the autofluorescence ability of some polymers to fluoresce upon excitation by mostly short-wavelength light without the need to incorporate any additional fluorochrome [24,25,26]. This ability is used in biomedical research and diagnosis, for example, to differentiate neoplasms and healthy tissue or cells [24]. Although autofluorescence has been studied in polymers since the 1980s, only few very recent studies demonstrate the possibility to use this phenomenon to detect plastic debris in sediment samples [27,28]. Polymer autofluorescence is a very attractive field of research for environmental studies, not only for the detection of MPs/NPs in environmental matrices but also for the study of the availability, absorption, and effects of environmentally relevant micro/nanoplastics on biota.

The autofluorescence of polyethylene terephthalate (PET) has been studied, mostly in order to identify the species formed during PET photo-oxidation or thermal degradation [29,30]. It has been reported that some intermolecular interactions may exist, generating states such as excimers. However, the nature of the fluorescence spectra of these polymers has not necessarily been elucidated thoroughly [24]. Fukazawa et al. [29] have hypothesized that crystalline and amorphous regions, and their boundary region, have a key role in photochemical and photophysical processes in polyethylene terephthalate powder. According to Itagaki et al. [31], PET has a fluorescent phenylene moiety in the main chain, showing its fluorescence peak at 330–340 nm in fluid solution. The fluorescence behavior of PET films has been reported to depend on whether the main-chain phenylene ring is in the crystalline or amorphous regions, which correspond to a fluorescence peak at about 330 nm or longer wavelengths, respectively [31].

The aim of this work is to investigate the autofluorescent potential of label-free model PET nanoplastics produced in the laboratory with a fast procedure optimized in a previous work [32]. Unlike the few works on mechanical milling reported in the literature [11,33,34], the novelty of the proposed protocol is based on the high-yield/high-speed mechanical fragmentation process used for the nanoplastic production, which has been tailored on the crystallinity and viscoelastic properties of the polyethylene terephthalate. In this way, the milling time is limited to 30 min in order to avoid any polymer overheating and, consequently, friction-induced thermal degradation or polymer agglomeration. Another advantage of the obtained model PET nanoplastics is due to the absence of dispersant agents or chemical solvents which could unpredictably affect the experiments on environmental systems.

**Scheme 1:**
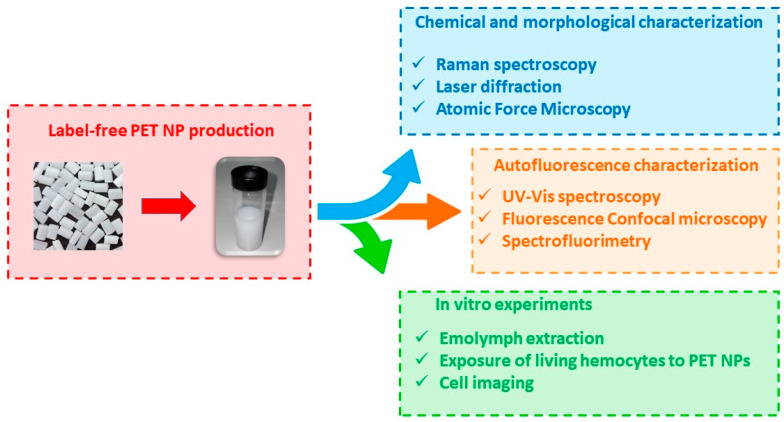
Schematic representation of the present study.

As reported in Scheme 1, model PET nanoparticles have been characterized from the chemical and morphological point of view and their autofluorescence has been analyzed by spectrofluorimetry and fluorescence microscopy. The emission spectra of label-free nanoplastics have been compared with those of nanoplastics labeled with Nile red, a well-recognized fluorescent dye for polymers. Finally, the suitability of label-free nanoplastics for biological studies has been assessed by in vitro exposure experiments with *Mytilus galloprovincialis* hemolymphatic cells in a time interval up to 6 h. The nanoplastic internalization into these cells, known to be provided with phagocytic activity [35,36], has been assessed by fluorescence microscopy.

## 2. Materials and Methods

### 2.1. Polyethylene Terephthalate Model Nanoplastics

**Scheme 2:**
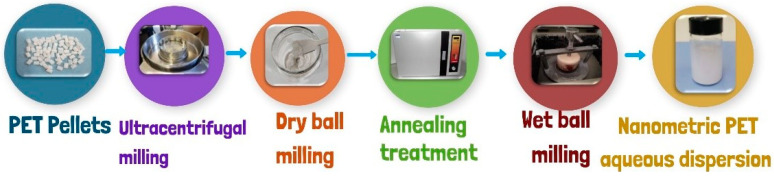
Schematic representation of the production steps of label-free PET NPs.

The model nanoplastics used in this work were produced starting from pellets of RT52 polyethylene terephthalate (PET) supplied by Invista Resins & Fibers GmbH (Gersthofen, Germany) through a procedure optimized in a previous work [32]. The starting polymer had an intrinsic viscosity of 0.634–0.638 dL/g, as reported in the technical datasheet. The pellets were characterized by an average diameter of 2.4 mm and length of 4 mm. An average crystallinity degree of 0.22 was measured on the as-received pellets by X-ray diffraction [32]. The production process of nanoplastics first involved a very-fast step of size reduction to the micrometric range by using a RETSCH ZM100 Ultra Centrifugal mill (RETSCH GmbH & Co., Düsseldorf, Germany) at 14,000 rpm (see Scheme 2). The micrometric powders are subjected to an annealing treatment at 160 °C for 4 h, followed by a slow cooling to room temperature. The annealing treatment has been demonstrated to be useful to erase the polymer amorphization arising by milling, and improve the milling effectiveness in reducing the particle size, avoiding particle agglomeration [32]. Then, in order to reduce the micrometric powders to the nanometer scale, a further comminution stage in a ball mill was necessary. The powders were wet-milled at 390 rpm for 30 min in a zirconia jar, using ultrapure water and zirconia balls in an ambient atmosphere and an S/1 1000B ball mill (Ceramic Instruments S.r.l, Sassuolo Modena, Italy) with parameters optimized in a previous work accounting for the structural changes in the semicrystalline polymer induced by milling [32]. Since the risk of contamination during preparation and analysis is very high [37], particular attention was taken to minimize MP and NP contamination during all the protocol steps and successive characterization activities.

The optimized method presented several advantages such as low cost, the absence of liquid nitrogen, the short production time, the high yield of the process, and the stability and reproducibility of the produced water-dispersed PET nanoparticles [32]. The obtained aqueous dispersion of label-free nanometric PET was then characterized, as reported below, in order to investigate any chemical modification and evaluate the morphology and size distribution. Then, it was used for the subsequent fluorescence and exposure studies.

For comparison purposes, fluorescent labeled PET nanoplastics were also produced using a novel procedure optimized in the present work. It was based on the labeling of PET microplastics with Nile red (CAS N. 7385-67-3) from Hello Bio Ltd (Dunshaughlin, Ireland), followed by their ball-milling up to the nanoscale. Nile red was chosen as a fluorescent dye, since its use in the analysis of microplastics has been proposed with different protocols by several researchers [38,39,40,41]. First of all, a stoke solution of Nile red (20 µg/mL) in acetone/ethanol (50:50 by volume) was prepared and stirred for 10 min at room temperature. PET microplastics were mixed with Nile red stock solution at a concentration of 10 mg/mL at 50 °C for 20 min. Then, the microplastics were rinsed three times with distilled water and filtered through a cellulose filter with pore size lower than 2 µm. The Nile-red-stained microplastics were then milled in a zirconia jar with zirconia balls according to the parameters already used for the production of label-free PET nanoplastics until the labeled particles reached the nanometer scale. At the end of this procedure, an aqueous dispersion of Nile-red-stained PET nanoplastics was finally obtained and used for the subsequent fluorescence and exposure studies.

### 2.2. PET Nanoplastic Characterization

The obtained PET nanoparticles were characterized by Raman spectroscopy with a LabRam confocal system (Horiba Jobin Yvon, Palaiseau, France), using a 100× objective. Excitation at 633 nm was provided by a He–Ne laser, delivering 7 mW at the sample surface. The results were the average of three replicates.

The size distribution of nanoparticles was measured by laser diffraction. A CILAS 1190 multi-laser particle size analyzer was used together with Particle Expert^®^ software (CPS Us, Inc., Madison, WI, USA). Samples were placed in a 500 mL water cell, lit by the low-intensity laser beam, and the scattered light was focused on the detectors by means of Fourier lens. The particle size measurement was based on the Mie theory for light scattering [42]. The results were the average of three replicates.

The morphology of the PET nanoplastics was characterized by atomic force microscopy (AFM), a technique still little-used in nanoplastic research. A MultiMode 8 AFM system (Bruker, Champs sur Marne, France) was used. The colloidal suspension of particles was diluted with ultrapure water, and 50 µL of the dispersion was spin-coated onto a steel disc with subsequent drying at room temperature. AFM imaging was carried out under ambient condition in the PeakForce Tapping mode using 0.01–0.025 Ω·cm antimony (n)-doped Si cantilevers RTESPA-300 (Bruker, nominal length 125 µm, nominal tip radius 8 nm). In PeakForce Tapping mode, the force curve describes the dependence of the interaction force between cantilever and specimen on the distance between them, and information about the surface topography can easily be extracted during the scan, without further post-processing [43]. The scanning parameters were optimized to obtain high-quality images of the surface topography. The scan sizes were set at 15 × 15 µm^2^, 10.4 × 10.4 µm^2^, 1.8 × 1.8 µm^2^, and 0.37 × 0.37 µm^2^, the scanning rates in the 0.30–0.45 Hz range, and the resolution at 640 lines per scan. Nanoscope Analysis v.1.5 (Bruker) software was applied for the AFM data processing, and for the size measurements.

A medium-pressure Hg UV lamp (UV HG 200 ULTRA, Jelosil Srl, Vimodrone, Italy) with maximum emission peak at 365 nm was used for assessing the fluorescence of microplastics and nanoplastics. Three 5 mL vials containing PET microplastic powders (with average size around 100 µm), the aqueous dispersion of PET nanoplastics, and distilled water were irradiated by the UV lamp.

The UV–Vis spectra of aqueous dispersions of label-free PET nanoparticles were measured with an Eon™ Spectrophotometer (BioTek, Winooski, VT, USA) in a standard 10 mm quartz cuvette. The absorption spectra were recorded in the range 200–400 nm.

The fluorescence emission spectra of aqueous dispersions of label-free PET nanoparticles were measured with a Cytation 5 multimode reader (BioTek, Winooski, VT, USA) in Corning^®^ 96-Well Black Polystyrene Microplates (Glendale, AR, USA) in the range 350–700 nm.

Fluorescence microscopic visualization of PET nanoplastics was performed using the Argon 488 nm and Elio-Neon 543 nm laser lines of a C1 NIKON confocal laser scanning unit coupled to a NIKON TE300 microscope with a 100X/1.30 oil objective (NIKON, Tokyo, Japan). Photomultiplier (PMT) and laser intensity settings and emission wavelengths were optimized for minimal bleed-through between the two channels.

### 2.3. In Vitro Exposure of Mytilus Galloprovincialis Hemocytes to PET NPs

Hemolymph was withdrawn from the adductor muscle of *Mytilus galloprovincialis* specimens (shell length 6.3 ± 1 cm, shell width 3.2 ± 0.5 cm; purchased from a local farm, Mare Vivo srl, Castro, Italy) using a syringe through a noninvasive method and diluted 1:1 in filtered seawater (0.22 µm filters). For each independent experiment (n = 3), the hemolymphs were sampled from 4 animals and pooled. A volume (50 µL) of diluted hemolymph was added to each well of a Corning^®^ 96-Well Black Polystyrene Microplate 96 and then incubated for 30 min at 18 °C to allow the cells to adhere to the well bottom. Then the excess hemolymph was gently removed and the adherent cells were exposed to 10 mg/L label-free PET NPs (100 µL) or 10 mg/L Nile-red-labeled PET NPs (100 µL) for 1, 2, 4, and 6 h at 16 °C. PET NPs and Nile-red-labeled PET NPs were suspended in filtered seawater. Control cells were incubated with 100 µL of filtered sweater, while no hemolymph was seeded into negative controls wells, only containing 100 µL of 10 mg/L label-free PET NPs or 10 mg/L Nile-red-labeled PET NPs. Four replicates per experimental condition were used.

The cells were visualized by video microscopy using a Cytation 5 imaging multi-mode reader (BioTek, Winooski, VT, USA) provided with 360/460 nm (blue), 485/528 nm (green), and 523/647 nm (red) fluorescence filter sets. All images were taken at 40× magnification. The concentrations of PET NPs used in the in vitro exposure experiments in this study were in agreement with the micro- and nanoplastic concentrations used in previous in vitro studies with *Mytilus galloprovincialis* [36] and represent the highest environmental levels of total MPs floating on the surface of the ocean gyres, where microplastics are known to be concentrated. Hemocyte viability was measured using the trypan blue staining method [44] under the course of the exposure experiments. A 0.4% trypan blue osmotically adjusted to the osmolarity of mussel hemolymph was used. High cell viability was observed under the course of the experiment, being about 89% after 6 h of experiment, and no significant differences between control and NP exposed cells were recorded.

## 3. Results

### 3.1. Nanoplastic Characterization

**Figure 1:**
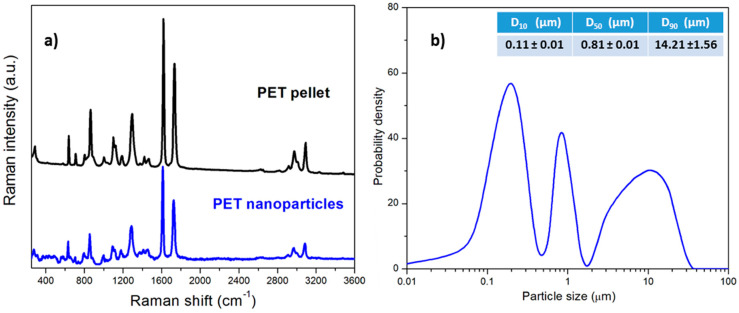
(**a**) Raman spectra of label-free PET nanoplastics and PET pellet; (**b**) particle size distribution of the aqueous dispersion of label-free PET nanoplastics.

The Raman spectra of pristine PET pellets and PET nanoplastics in Figure 1a present the most characteristic vibrational peaks of polyethylene terephthalate structure at 1611 cm^−1^, assigned to C=C stretching of aromatic rings, and at 1725 cm^−1^, attributed to the carbonyl C=O stretching [45,46,47,48]. Since no differences are detectable between the two samples and the spectrum of PET nanoplastics does not display any shoulder or peak associated with carboxylic acid groups arising from PET oxidation in the range 1680–1700 cm^−1^ [49], it can be concluded that the mechanical fragmentation process used for the production of PET nanoplastics does not cause any chemical modification of the produced PET nanoplastics.

The laser diffractometric analysis shown in Figure 1b reveals a polydisperse size distribution of the label-free nanoplastics. A significant nanometric fraction is centered at about 200 nm, while a sub-micrometric and micrometric fraction are centered, respectively, at about 0.8 µm and 10 µm. The average sizes at 10% (D10), 50% (D50), and 90% (D90) by volume of the analyzed particles are 0.11 µm, 0.81 µm, and 14.21 µm, respectively. Compared to commercial nanoplastics, which are generally characterized by monomodal size distribution, the observed size heterogeneity makes the produced model nanoplastics more environmentally relevant and, therefore, more similar to the nanoplastics that could be sampled in the marine environment [1,33,38]. Ter Halle et al. [17] and Gigault et al. [50] observed the presence of several populations of highly polydisperse nanoparticles in the 1–1000 nm range among the debris collected in the North Atlantic Subtropical Gyre. The NPs in the environment are slowly created by the degradation of primary and secondary microplastics, mostly due to the combined effect of photodegradation and mechanical abrasion with sand [51]. One can consider that the mechanical abrasion is a sort of ball-milling process where small hard sand granules play the role of zirconia balls used in the laboratory experiments. Additional degradation mechanisms producing chemical changes in the polymer, due to photodegradation leading to possible presence of end groups on the NP surface, can occur. However, the entity of this phenomenon is already under discussion [2].

**Figure 2:**
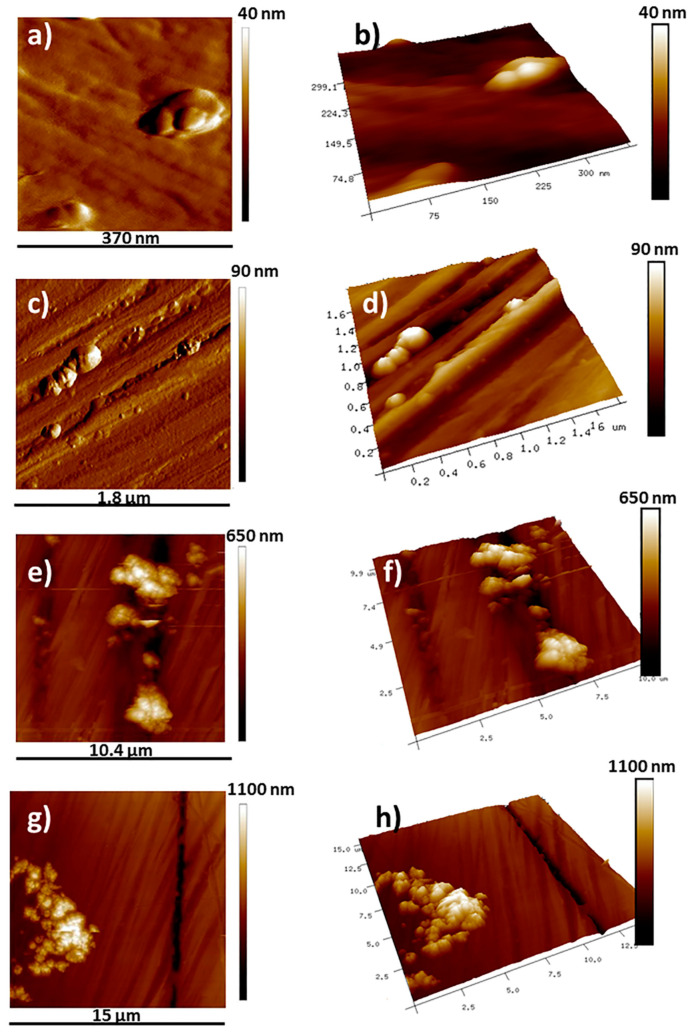
Topographic 2D and 3D AFM images of label-free PET nanoparticles at different scan areas: 0.37 × 0.37 μm^2^ (**a**,**b**); 1.8 × 1.8 μm^2^ (**c**,**d**); 10.4 × 10.4 μm^2^ (**e**,**f**); and 15 × 15 μm^2^ (**g**,**h**).

The size heterogeneity of the prepared PET nanoplastics has been confirmed by the topographic AFM images presented in Figure 2 in a color scale indicating the Z height. For each row in Figure 2, the images on the left side are two-dimensional topographic scans while those on the right side are three-dimensional (3D) AFM scans for a better detection of the nanoparticles sizes and shapes. Several images have been taken from different locations and with different image sizes, but only the most representative are reported in Figure 2. From AFM images, it results that the obtained nanoplastics are not spherical, unlike the commercial ones, but characterized by an elongated shape. For example, nanoparticles with length between 87 nm and 150 nm and height lower than 40 nm are observed in Figure 2a,b, while the particles in Figure 2c,d present lengths in the range 160–710 nm and heights lower than 90 nm. Bigger particles and aggregates with lengths and heights in the sub-micrometric and micrometric range are reported in Figure 2e–h.

AFM results on the label-free nanoplastics show the polymorphic nature of the label-free nanoplastics characterized by irregular shapes and surfaces. Therefore, the prepared PET nanoparticles are able to mimic the irregularity of size, shape, and surface, which characterizes microplastics and nanoplastics present in the marine environment [52]. Finally, the results in Figure 2 also confirm the high potential of AFM to study nanoplastic morphology. Although AFM is still a very-little-used technique in this field, it provides images with high resolution in the nanometric scale, can be used to investigate the surface of non-conducting polymers, as in the case of MPs and NPs, and provides direct 3D images of the surface structure of the polymers.

### 3.2. Nanoplastic Autofluorescence

**Figure 3:**
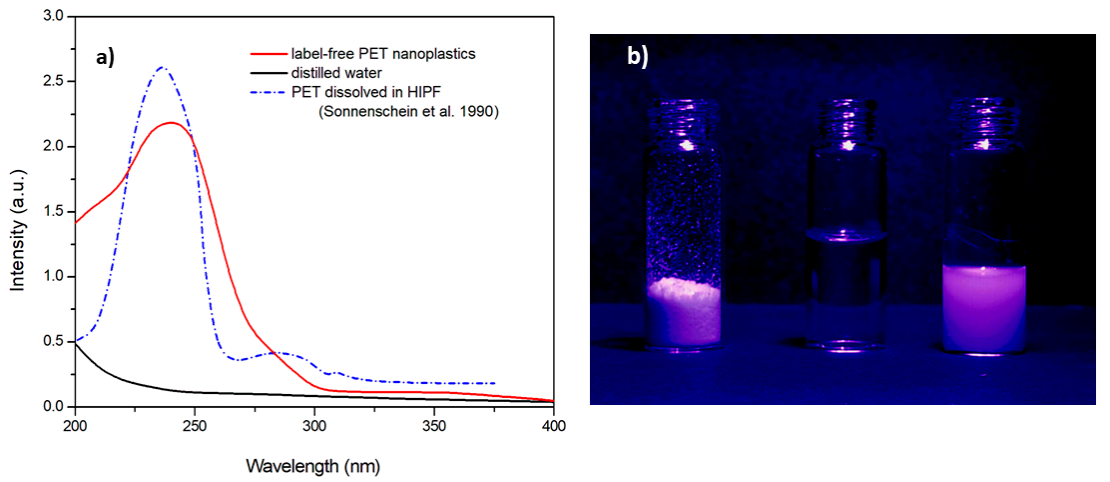
(**a**) UV–Vis absorption spectra of the aqueous dispersion of label-free PET nanoparticles, distilled water, and, for comparison purposes, the references spectrum of PET dissolved in hexafluoroisopropanol (HIPF) reported by Sonnenschein et al. [53]. (**b**) Fluorescence of label-free PET microplastics (left vial) and label-free nanoplastic dispersion (right vial) under UV irradiation at 365 nm (distilled water in the center vial).

The aqueous dispersion of label-free PET NPs presents a strong absorption in the UV spectrum with a peak centered at 240 nm, as shown in Figure 3a. This result is in agreement with the literature data on PET dissolved in solvents, as in hexafluoroisopropanol (HIPF) reported by Sonnenschein et al. [53], indicating that the mechanical fragmentation process for nanoplastic production has not changed the optical properties of PET nanoparticles.

When exposed to the UV irradiation of a lamp, both the label-free microplastics and nanoplastics exhibit fluorescence, as can be observed in Figure 3b, where a vial containing distilled water has been used as a reference. The fluorescence of nanoplastic dispersion has been therefore studied by specrofluorimetric analysis.

**Figure 4:**
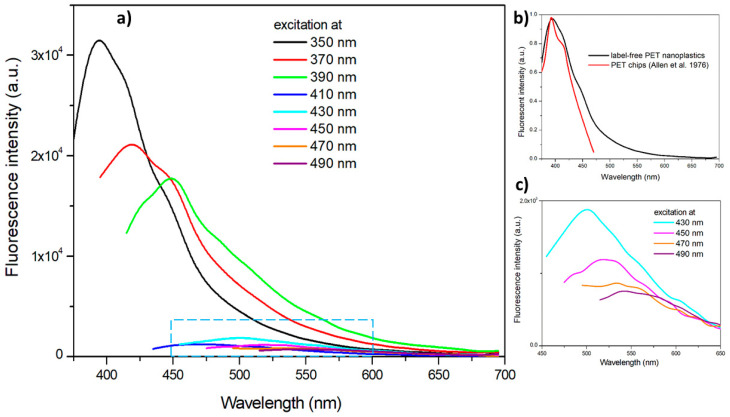
(**a**) Fluorescence emission spectra: (**a**) label-free PET nanoparticles dispersed in distilled water at different excitation wavelengths; (**b**) comparison of emission spectra at 350 nm excitation of label-free PET nanoparticles and PET chips reported by Allen et al. [54]; (**c**) magnification of the spectra area hatched in light blue, referring to emission spectra of label-free PET nanoparticles excited in the 430–490 nm range.

The dependence of the emission fluorescent spectra of label-free PET NPs on the excitation wavelength is reported in Figure 4a. It is clearly observable the decrease of the fluorescence intensity with the excitation wavelength in the range between 350 nm and 490 nm, which has been previously attributed to the formation of dimers due to intramolecular interactions of non-nearest-neighbor phenyl rings or intermolecular interactions between adjacent chains resulting in dimers [54,55]. The spectrofluorimetric results confirm the florescence behavior shown in Figure 3b. In fact, by exciting the label-free nanoplastics at 365 nm (nominal central frequency of the UV lamp), the emission peak should be around 405 nm, therefore in the violet range, as detectable in Figure 2b.

The fluorescence spectrum of label-free PET nanoparticles excited at 350 nm is compared in Figure 4b with that reported by Allen et al. [54] in a study on PET photodegradation. The emission curve of PET nanoparticles, presenting a peak at 390 nm and a shoulder at 410 nm, displays a very good agreement with the spectrum reported by Allen et al. [54]. This is further evidence that the method optimized for the production of model PET nanoplastics does not change the fluorescence properties of the polymers, providing fluorescence ability also at the nanoscale level.

**Figure 5:**
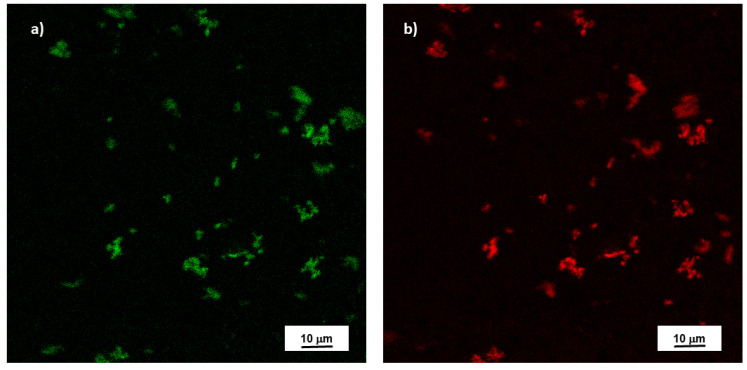
Representative fluorescence of label-free NPs visualized by the Argon 488 nm (**a**) and Elio-Neon 543 nm (**b**) laser lines of a C1 NIKON confocal laser scanning unit coupled to a NIKON TE300 microscope with a 100X/1.30 oil objective.

According to the fluorescence emission spectra displayed in Figure 4a, the maximum emission intensity in the excitation range used for the measurements is reached at excitation of 350 nm. At excitation wavelengths higher than 410 nm, the intensities are significantly lower but the fluorescence intensity displays a maximum at wavelength higher than 500 nm, as reported by the magnification of the spectra area hatched in light blue in Figure 4c. This is also confirmed by fluorescence microscopy images reported in Figure 5. Label-free nanoplastics are clearly observable in the confocal fluorescence microscopy under the green and red fluorescence.

### 3.3. In Vitro Exposure of Mytilus Galloprovincialis Hemolymphatic Cells to Label-Free PET NPs

The spectrofluorimetric and fluorescence microscopic results have been exploited to check if label-free model PET nanoplastics can be used in ecotoxicological studies addressed to investigate nanoplastics interactions with biological systems. In the present work, the hemolymphatic cells of the bivalve mollusk *Mytilus galloprovincialis* were used as model target cells and were in vitro exposed to label-free NPs and, for comparison, to Nile-red-labeled NPs for a time span of 6 h. Bivalve mollusks are widely utilized as bioindicator organisms in coastal and marine biomonitoring and assessment [56,57]. Their high water filtration rate due to their filter-feeding nature makes these organisms particularly vulnerable to the exposure to micro/nanoplastics [58,59,60]. Recent literature reports that polyethylene terephthalate (PET) is one of the most common polymers in *Mytilus galloprovincialis* in the Adriatic Sea [61,62]. Hemolymph, being the internal circulating body fluid of these organisms, is responsible for the pollutant transport and distribution to the whole body. In particular, hemolymphatic cells, being involved in the internal defense system of the organisms, are known to be provided with phagocytic activity [35], whose defensive function accounts for elimination of non-self-components from the body. Nanoparticle endocytosis by mussel hemolymphatic cells has been very recently demonstrated with fluorescent-labeled polystyrene nanoparticles of different sizes, from 50 nm to 1 µm [36]. Therefore, hemolymphatic cells represent a good model for studies addressing NPs cellular uptake.

**Figure 6:**
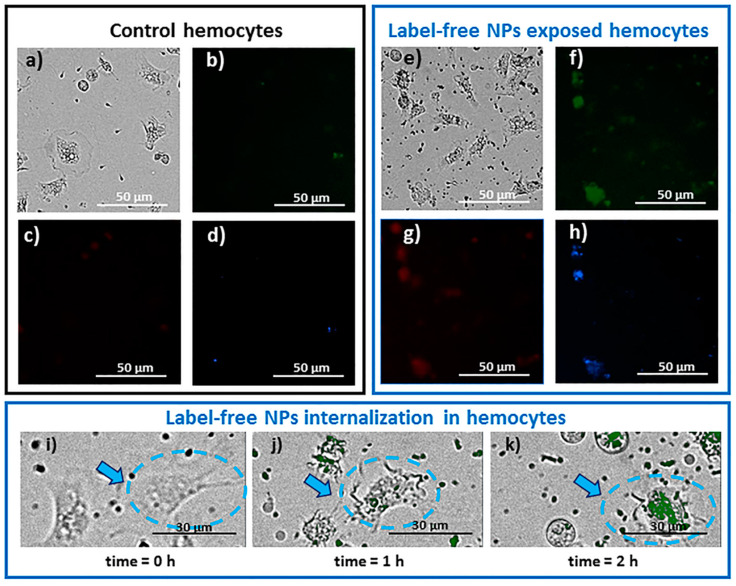
Representative images of control (**a**–**d**) mussel hemocytes and label-free NPs (10 mg/mL) exposed mussel hemocytes (**e**–**h**) after 2 h exposure. The cells were imaged by video microscopy using a Cytation 5 imaging multi-mode reader provided with 360/460 nm (blue), 485/528 nm (green), and 523/647 nm (red) filter sets. All images were taken at 40× magnification; (**i**–**k**) representative images of mussel granulocytes exposed to 10 mg/mL label-free NPs and observed in the same ROI (region of interest). The arrow indicates the same granulocyte observed at different times (scale bar 30 µm). The green signal of NPs has been merged with the bright-field image.

The internalization of label-free NPs into the *Mytilus galloprovincialis* hemolymphatic cells was visualized by live cells fluorescence imaging. Cells internalizing label-free PET NPs display a detectable fluorescence at the 360/460 nm (blue), 485/528 nm (green), and 523/647 nm (red) filter sets of the Cytation 5 imaging multi-mode reader used for the visualization. The typical appearances of control and treated cells after 2 h exposure to label-free PET nanoplastics as observed under fluorescence microscopy are reported in Figure 6a–h. The NP internalization by cells is clearly detectable as fluorescence spots in some cells, mostly in the green and blue fluorescence. A very slight native background autofluorescence of the cells is observed, mostly in the green and blue fluorescence (Figure 6b,d). As reported in the literature, it has been ascribed to the presence of lipofuscins and cytochromes [63].

The internalization of label-free NPs into the *Mytilus galloprovincialis* hemolymphatic cells can be better visualized by representative timelapse video microscopy images of the same mussel granulocytes exposed to 10 mg/L label-free NPs with the green signal of NPs merged with the bright-field image (see Figure 6i–k). A time-dependent increase in the fluorescence signal inside the cells can be observed. Moreover, the cells show a detectable change of their shape during the course of the exposure, with a tendency to rounding and retraction of some processes.

**Figure 7:**
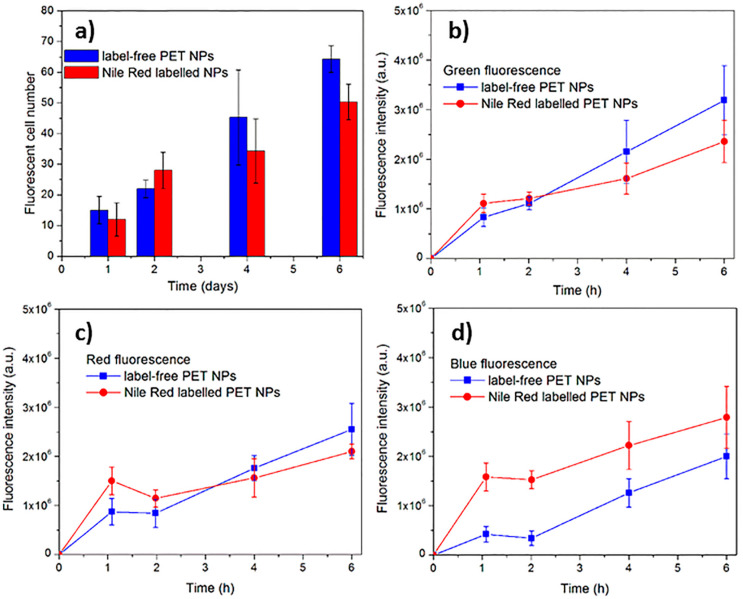
(**a**) Number of *M. galloprovincialis* hemolymphatic cells internalizing nanoparticles observed during the exposure to label-free PET NPs (10 mg/L) or Nile-red-labeled PET NPs (10 mg/L) for a time span of 6 h; (**b**–**d**) fluorescence intensity during in vitro exposure of *M. galloprovincialis* hemocytes to label-free and Nile-red-labeled NPs (10 mg/L) under the green excitation at 485 nm (**b**), the red excitation at 523 nm (**c**), and blue excitation at 360 nm (**d**).

After having detected internalization of label-free PET NPs into mussel hemocytes, the phenomenon was quantified by fluorescence microscopy, carried out at time intervals during a 6 h exposure experiment. Figure 7a reports the number of cells internalizing fluorescent nanoparticles recorded in the observation area (300 µm^2^) at different times. The slight native background autofluorescence of the cells was subtracted from the fluorescence intensity values reported in the graphs. A time-dependent increase of the number of the internalizing cells was observed. After 4 h and 6 h exposure, the number of cells internalizing label free NPs is about three and four times higher with respect to the value observed after 1 h, respectively. The statistical analysis of data performed by two-way ANOVA confirms a significant (*p* < 0.001) increase in the number of fluorescent cells with the time of exposure, but no significant differences are detected between free-label PET NPs and Nile-red-labeled NPs.

Figure 7b–d show the quantification of the fluorescence intensity of the cells, after subtracting background at different observation times during the course of a 6 h exposure experiment. Under the green excitation at 485 nm, there is no significant difference among the fluorescence intensities of the cells exposed to label-free NPs and those exposed to Nile-red-labeled NPs, as confirmed by two-way ANOVA. This proves the suitability of the autofluorescent label-free NPs to be used in biological experiments. Concerning the blue excitation, the fluorescent intensities of cells exposed to Nile-red-labeled NPs is always higher than that of the cells exposed to label-free NPs (*p* < 0.001, two-way ANOVA). Despite the superiority of the blue fluorescent signal associated with Nile-red-labeled NPs, this does not prevent the possibility to use the label-free NPs, which are, however, detectable in the green and blue excitation (see Figure 6f–k).

The assessment of the label-free PET NPs internalization into hemocytes and the observation of cell rounding and loss of pseudopods, which are indicative of stress in exposed cells, suggest that hemocytes represent one of the first targets of potential PET NP effects in mussels. Considering the important role played by immune cells in the physiology of the organisms, any impairment of immune cell function could be reflected in alterations of the health status of the whole organism. It is known that the exposure to different types of labeled NPs is able to produce changes in hemocytes motility, apoptosis, ROS, and phagocytic capacity [36,64]. Moreover, PET nanoparticles have been recently demonstrated to induce reduction of cell proliferation, increase of intracellular reactive oxygen species (ROS) concentrations, and changes in gene expression in RAW 264.7 cells, a macrophage-like murine cell line [20].

Therefore, the results obtained in the present study with label-free PET NPs, similar to those found in the marine environment, open the perspective for future in vitro and in vivo studies addressing the absorption, tissue distribution, and the potentially toxic effects of PET nanoparticles on aquatic organisms and humans in the framework of environmental risk assessment.

## 4. Conclusions

A top-down approach based on mechanical fragmentation has enabled to produce model PET nanoparticles with polydisperse size and irregular shape and surface by means of a process close to the mechanical abrasion of microplastics by small sand granules occurring in the aquatic environment. In addition to the environmental relevance, they are also characterized by the absence of dispersant agents or chemical solvents which could unpredictably affect the experiments on living systems, leading to different and contradictory results.

The main result of the present study is the autofluorescence of the produced label-free PET NPs, which have shown comparable fluorescent properties with PET NPs labeled with Nile red. This demonstrates their suitability for biological studies, which has been confirmed by in vitro exposure experiments with *Mytilus galloprovincialis* hemolymphatic cells in a time interval up to 6 h. The observed internalization of label-free NPs into hemocytes and the observation of cell rounding and loss of pseudopods, which are indicative of stress in exposed cells, suggest that hemocytes represent one of the first targets of potential PET NP effects in mussels.

The results of the autofluorescence study on model PET nanoplastics provide an innovative approach for plastic risk assessment. Implications for future biological studies can arise from the several advantages associated with label-free nanoparticles, such as the lack of any additional toxicity arising from the fluorescent dye and its possible leaching during long-time experiments, and their high resistance to photobleaching during microscopic experiments.

## Data Availability

Data sharing is not applicable to this article.
